# Data on contents of fifty phenolic compounds in three rivers in Tianjin, China

**DOI:** 10.1016/j.dib.2018.03.005

**Published:** 2018-03-09

**Authors:** Wenjue Zhong, Donghong Wang, Zijian Wang

**Affiliations:** a Tianjin Key Laboratory of Environmental Remediation and Pollution Control, Key Laboratory of Pollution Processes and Environmental Criteria of Ministry of Education, College of Environmental Science and Engineering, Nankai University, Tianjin 300350, China; b Key Laboratory of Drinking Water Science and Technology, Research Center for Eco-Environmental Sciences, Chinese Academy of Sciences, Shuangqing Rd 18. Haidian District, Beijing 100085, China; c State Key Laboratory of Environmental Aquatic Chemistry, Research Center for Eco-Environmental Science, Chinese Academy of Sciences, Beijing 100085, China

**Keywords:** Phenolic compounds, Suspended particulate matter, Surface water, Sediment, Tianjin

## Abstract

This article contains data related to the research article entitled “Distribution and potential ecological risk of 50 phenolic compounds in three rivers in Tianjin, China” [1]. This data article reports the detailed information for the contaminant level of phenolic compounds in three rivers in Tianjin, China. The data collects from seven sample sites in Beitang drainage river, sixteen sample sites in Dagu drainage river, and fourteen sample sites in Yongdingxin river. The ranges, standard deviations, average values, median values of the concentrations of identified phenolic compounds in three rivers and the standard deviations, average values, the maximum values of risk quotients of identified phenolic compounds in three rivers are listed in this paper.

**Specifications table**

**Table:** 

Subject area	Environmental Science
More specific subject area	Phenolic pollutants in river
Type of data	Tables
How data was acquired	Phenolic compounds measurement was carried out using gas chromatography (Gas Chromatography (GC): 6890A, Agilent Technologies, Inc.) coupled with mass spectrometry (Mass Spectrometry (MS): 5975C, Agilent Technologies, Inc.)
Data format	Raw, analyzed
Experimental factors	The data were obtained in two season, wet-season and dry-season, and all suspended particulate matter sample, surface water sample and sediment sample were measured for each sample site.
Experimental features	Retention time locking (RTL) technology and deconvolution reporting software (DRS) were used to determine the contents of phenolic compounds.
Data source location	Tianjin, China
Data accessibility	Data provided in the article is accessible to the public.
Related research article	“Distribution and potential ecological risk of 50 phenolic compounds in three rivers in Tianjin, China” (in press).

**Value of the data**

• The data provide more details on distribution of phenolic compounds in rivers in Tianjin.
• The data present here will be valuable for ecological risk assessment of phenolic compounds in water environment.
• The data can be used for the water environmental managers for proper operation.

## 1 Data

The detailed information of 50 phenolic compounds are displayed in our previously papers [2], [3], [4]. The concentration ranges, standard deviations, average concentrations, and median values for each of identified phenolic compound in three rivers are listed in the Table 1, Table 2, Table 3, Table 4, respectively. Table 4 described the risk quotient of identified phenolic compounds in surface water in three rivers. The risk quotient was defined as the ratio of predicted environmental concentration (PEC) to the predicted no-effect concentration (PNEC).

**Table 1:** The concentrations of identified phenolic compounds in Beitang drainage river.

		Wet-season			Dry-season		
		Average	Median	Average±Std	Average	Median	Average±Std
SPM (µg/kg)	Phenol	14.2	4.79	14.2 ± 28.2	1.33	–	1.33 ± 2.98
	2-cresol	0.63	–	0.63 ± 1.55			
	3-cresol	0.83	–	0.83 ± 1.5			
	4-cresol	1.13	–	1.13 ± 2.78	0.20	–	0.2 ± 0.45
	2,4-xylenol	1.30	1.13	1.3 ± 1.32			
	2,5-dichlorophenol	0.51	–	0.51 ± 1.25			
	2-naphthol				0.36	–	0.36 ± 0.56
	p-chloro-m-xylenol	2.35	0.96	2.35 ± 4.05			
	2,4-dichloro-3-ethyl-6-nitrophenol	0.87	1.28	0.87 ± 0.77	0.34	–	0.34 ± 0.49
Surface water (µg/L)	Phenol	2.43	0.01	2.43 ± 3.86	3.58	0.18	3.58 ± 4.9
	2-cresol	15.0	3.39	15.0 ± 18.9	4.75	3.73	4.75 ± 5.22
	3-cresol	4.93	1.30	4.93 ± 6.62	4.01	0.23	4.01 ± 6.12
	4-cresol				3.10	–	3.1 ± 4.9
	2,4-xylenol	6.24	1.59	6.24 ± 11.1	11.1	9.54	11.1 ± 10.1
	4-nitrophenol	0.43	–	0.43 ± 0.7			
	2,6-dichlorophenol				1.44	1.51	1.44 ± 1.18
	2,4-dichlorophenol				1.56	1.64	1.56 ± 1.27
	2,5-dichlorophenol	0.35	–	0.35 ± 0.85	2.15	2.25	2.15 ± 1.76
	p-chloro-m-xylenol	0.65	–	0.65 ± 1.27	0.68	0.82	0.68 ± 0.52
	2-Biphenylol	0.20	0.22	0.2 ± 0.19			
	2-sec-Butylphenol	1.59	–	1.59 ± 3.88	2.45	2.17	2.45 ± 2.3
	2-naphthol	5.41	3.65	5.41 ± 4.91			
	Pyrocatechol				0.04	–	0.04 ± 0.09
	4-chlorophenol	0.10	–	0.1 ± 0.17			
	2,3,6-Trimethylphenol	0.57	–	0.57 ± 0.75			
	2,4-dichloro-3-ethyl-6-nitrophenol	0.41	0.38	0.41 ± 0.35			
Sediment (µg/kg)	Phenol	0.82	0.58	0.82 ± 0.85	0.13	–	0.13 ± 0.28
	2-cresol	8.05	–	8.05 ± 17.16			
	3-cresol	2.87	–	2.87 ± 5.93			
	4-cresol	4.01	1.72	4.01 ±5.54			
	2-chlororphenol	1.62	–	1.62 ± 3.62			
	2,4-xylenol	3.29	4.73	3.29 ± 2.34			
	Pyrocatechol	0.56	–	0.56 ± 1.26			
	Resorcinol	0.37	–	0.37 ± 0.84			
	2,5-dichlorophenol				0.09	0.06	0.09 ± 0.09
	2-naphthol	1.48	1.20	1.48 ± 1.71			
	Hexanoes	33.2	–	33.2 ± 74.2	8.80	–	8.8 ± 19.7
	4-chlororphenol	0.18	–	0.18 ± 0.27	0.05	–	0.05 ± 0.11
	2-sec-Butylphenol	0.20	–	0.2 ± 0.28	0.003	–	0 ± 0.01
	2,4-dichloro-3-ethyl-6-nitrophenol	1.50	1.39	1.5 ± 1.51			

SPM: suspended particulate matter; std.: standard deviations; –: the detection frequencies of total phenolic compounds were lower than 50%, so that the median value could not be calculated.

**Table 2:** The concentrations of identified phenolic compounds in Dagu drainage river.

		Wet-season			Dry-season		
		Average	Median	Average±Std	Average	Median	Average±Std
SPM (µg/kg)	Phenol	50.0	5.51	50 ± 105	1.30	–	1.3 ± 1.63
	2-cresol	1.81	–	1.81 ± 4.1			
	3-cresol	1.65	–	1.65 ± 3.14			
	4-cresol	4.35	2.00	4.35 ± 5.95			
	2-chlororphenol	2.13	–	2.13 ± 8.53			
	2,4-xylenol	13.1	–	13.1 ± 39.4			
	2,4-dichlorophenol	2.52	–	2.52 ± 6.89			
	2-nitrophenol	3.56	–	3.56 ± 10.9			
	2,4,6-Trichlorophenol	1.25	–	1.25 ± 5			
	2-naphthol	43.1	12.6	43.1 ± 69.2	1.28	1.14	1.28 ± 1.44
	Hexanoes	4.17	4.18	4.17 ± 4.07	2.66	2.43	2.66 ± 2.94
	Pyrocatechol				1.20	–	1.2 ± 3.4
	2,4-dichloro-3-ethyl-6-nitrophenol	0.97	–	0.97 ± 2.19			
Surface water (µg/L)	Phenol	0.91	–	0.97 ± 3.88	2.21	–	2.21 ± 4.81
	2-cresol	0.82	–	0.87 ± 1.94	0.21	–	0.21 ± 0.59
	3-cresol	20.0	1.86	21.3 ± 66.4			
	4-cresol	21.1	3.44	22.4 ± 66.9	0.33	–	0.33 ± 0.93
	2,4-xylenol	5.43	–	5.77 ± 22.6			
	2,4-dichlorophenol	0.09	–	0.1 ± 0.39			
	2-nitrophenol	1.11	–	1.18 ± 4.39			
	4-nitrophenol	0.11	–	0.12 ± 0.49			
	2-sec-Butylphenol	0.09	–	0.1 ± 0.4			
	2-naphthol	288	267	305 ± 219	3.45	–	3.45 ± 4.03
	4-chlororphenol	0.07	–	0.08 ± 0.3			
	2,3,6-Trimethylphenol	3.11	–	3.31 ± 8.25	0.12	–	0.12 ± 0.35
	2,5-dichlorophenol	0.08	–	0.08 ± 0.34			
	p-chloro-m-xylenol	9.95	–	10.6 ± 39.1			
Sediment (µg/kg)	Phenol	0.82	–	0.82 ± 1.87			
	2-cresol	115	–	115 ± 439			
	3-cresol	37.6	–	37.6 ± 147			
	4-cresol	36.9	–	36.9 ± 141			
	Pentachlorophenol	4.07	–	4.07 ± 16.3	1.71	–	1.71 ± 4.83
	2-naphthol	7.49	–	7.49 ± 13.2			
	Hexanoes	12.1	10.4	12.1 ± 12.3	11.00	–	11.0 ± 29.6
	2,3,6-Trimethylphenol				0.29	–	0.29 ± 0.55
	2-nitrophenol				0.18	–	0.18 ± 0.5
	2-sec-Butylphenol	1.09	–	1.09 ± 2.99	0.27	–	0.27 ± 0.77
	3,4,5-Trichlorophenol	0.18	–	0.18 ± 0.72			

SPM: suspended particulate matter; std.: standard deviations; –: the detection frequencies of total phenolic compounds were lower than 50%, so that the median value could not be calculated.

**Table 3:** The concentrations of identified phenolic compounds in Yongdingxin River.

		Wet-season			Dry-season		
		Average	Median	Average±Std	Average	Median	Average±Std
SPM (µg/kg)	Phenol	10.7	8.02	10.7 ± 13.1			
	4-cresol				0.81	–	0.81 ± 3.03
	Pyrocatechol				2.40	–	2.4 ± 4.9
	Hexanoes	2.32	–	2.32 ± 3.31	3.27	3.40	3.27 ± 3.03
Surface water (µg/L)	Phenol				1.23	–	1.23 ± 4.04
	2-cresol	0.11	–	0.11 ± 0.36			
	2,4-xylenol	0.49	–	0.49 ± 1.76	0.27	–	0.27 ± 1
	Pyrocatechol				0.18	–	0.18 ± 0.66
	2-nitrophenol				0.24	–	0.24 ± 0.9
	4-nitrophenol				0.10	–	0.1 ± 0.37
	2-naphthol	0.63	–	0.63 ± 1.37			
Sediment (µg/kg)	2-cresol	0.89	–	0.89 ± 2.87			
	2-naphthol	0.59	–	0.59 ± 1.19			
	Hexanoes	3.64	–	3.64 ± 5.23	0.34	–	0.34 ± 0.89

SPM: suspended particulate matter; std.: standard deviations; -: the detection frequencies of total phenolic compounds were lower than 50%, so that the median value could not be calculated.

**Table 4:** The risk quotient of identified phenolic compounds in surface water in three rivers (average ± std.).

Chemicals	BDR		DDR		YDXR		PNEC μg/L
	Wet-season	Dry-season	Wet-season	Dry-season	Wet-season	Dry-season	
Phenol	0.13 ± 0.22 (<0.54)	0.19 ± 0.28 (<0.57)	0.05 + 0.2 (<0.82)	0.12 + 0.27 (<0.79)		0.06 + 0.21 (<0.80)	19
2- cresol	1.25 ± 1.7 (<4.38)	0.4 ± 0.48 (<1.30)	0.07 + 0.16 (<0.45)	0.02 + 0.05 (<0.16)	0.01 + 0.03 (<0.11)		12
3- cresol	0.41 ± 0.6 (<1.56)	0.33 ± 0.56 (<1.37)	1.77 + 5.53 (<22.4)				12
4-cresol		0.26 ± 0.45 (<1.09)	1.86 + 5.57 (<22.7)	0.03 + 0.08 (<0.25)			12
2,4-xylenol	0.8±1.53 (<4.21)	1.43 ± 1.41 (<3.48)	0.74 + 2.9 (<11.6)		0.06 + 0.23 (<0.85)	0.03 + 0.13 (<0.48)	7.8
4-chlorophenol	0.01±0.01 (<0.34)		0.01 + 0.02 (<0.09)				13
2,5-dichlorophenol	0.04±0.11 (<0.29)	0.25 ± 0.23 (<0.54)	0.01 + 0.04 (<0.16)				8.5
2,4-dichlorophenol		0.18 ± 0.16 (<0.39)	0.01 + 0.05 (<0.18)				8.5
2,6-dichlorophenol		0.17 ± 0.15 (<0.36)					8.5
4-nitrophenol	0.02 ± 0.04 (<0.098)		0.01 + 0.03 (<0.11)			0.01 + 0.02 (<0.08)	18
2-nitrophenol			0.07 + 0.24 (<0.98)			0.01 + 0.05 (<0.18)	18
2,3,6-Trimethylphenol	0.11 ± 0.16 (<0.38)		0.66 + 1.65 (<4.94)	0.02 + 0.07 (<0.22)			5
p-chloro-m-xylenol	0.13 ± 0.26 (<0.71)	0.13 ± 0.11 (<0.26)	2.03 + 7.51 (<30.2)				5.2
2-naphthol	0.64 ± 0.62 (<1.93)		36.0 + 25.8 (<63.5)	0.41 + 0.5 (<1.22)	0.07 + 0.16 (<0.54)		8.5
Pyrocatechol		0 ± 0 (<0.007)				0 + 0.02 (<0.07)	36
2-Biphenylol	0.04 ± 0.04 (<0.09)						5.4
2-sec-Butylphenol	0.4 ± 1.05 (<2.76)	0.61 ± 0.63 (<1.65)	0.03 + 0.1 (<0.40)				4
2,4-dichloro-3-ethyl-6-nitrophenol	0.08 ± 0.08 (<0.21)						2.8

BDR: Beitang drainage river; DDR: Dagu drainage river; YDXR: Yongdingxin River;

(): The numbers in bracket are the maximum values of risk quotients of identified phenolic compounds.

PNEC: the predicted no-effect concentration (μg/L).

## 2 Experimental design, materials and methods

### 2.1 Design

Thirty-seven sample sites were set in three rivers. b1–b7 were situated in Beitang drainage river (BDR), d1–d16 were located in Dagu drainage river (DDR) and y1–y14 were located in Yongdingxin river (YDXR). Thirty-seven surface water samples, thirty-seven SPM samples and thirty-six sediment samples (excluding b6) were collected in the wet season. Twenty-nine surface water samples, suspended particulate matter (SPM) samples and sediment samples were collected in dry season (excluding b3, d2, d4, d6, d9-d11, d14).

### 2.2 Materials

The pesticide-residue grade n-hexane and dichloromethane were purchased from Mallinchrodt Baker, Inc. (USA). Derivatization reagent N,O-bis(trimethylsilyl)trifluoroacetamide (BSTFA) with 1% trimethylchlorosilane (TMCS) was purchased from Supelco Co. (USA). Glass fiber filter membranes were purchased from Millipore Co. The C18 cartridges (500 mg, 6 mL) and Oasis HLB cartridges (500 mg, 6 mL) were purchased from Supelco Co. and Waters (USA), respectively.

### 2.3 Pre-treatment and analysis process

The chemicals and materials used to treat and analyze samples, the detailed methods for preparing water samples and analytical procedures have been published elsewhere [1], [2], [3], [4].
