# Supplementary material for: Data on contents of fifty phenolic compounds in three rivers in Tianjin, China
Source: Data Brief. 2018 Mar 9;18:124–30. doi: 10.1016/j.dib.2018.03.005 (PMC5996147; doi:10.1016/j.dib.2018.03.005)
Supplement: Supplementary file 1 — Supplementary material. [file mmc1.docx]

There are [no](https://www.baidu.com/s?wd=no&tn=44039180_cpr&fenlei=mv6quAkxTZn0IZRqIHckPjm4nH00T1d9PWT1nhm3Pjb3PHwWnHnv0ZwV5Hcvrjm3rH6sPfKWUMw85HfYnjn4nH6sgvPsT6KdThsqpZwYTjCEQLGCpyw9Uz4Bmy-bIi4WUvYETgN-TLwGUv3EnHRYnjbsPHbYrjmLrjD1PWn3Ps) conflicts of interest
